# Early-Onset Efficacy and Safety Pilot Study of Amphetamine Extended-Release Oral Suspension in the Treatment of Children with Attention-Deficit/Hyperactivity Disorder

**DOI:** 10.1089/cap.2018.0078

**Published:** 2019-01-28

**Authors:** Ann C. Childress, Judith C. Kando, Thomas R. King, Antonio Pardo, Barry K. Herman

**Affiliations:** ^1^ Center for Psychiatry and Behavioral Medicine, Inc., Las Vegas, Nevada.; ^2^ Tris Pharma, Inc., Monmouth Junction, New Jersey.

**Keywords:** ADHD, attention-deficit/hyperactivity disorder, amphetamines, extended-release oral suspension, efficacy, early onset

## Abstract

***Objective:*** To determine whether amphetamine extended-release oral suspension (AMPH EROS) has an onset of effect at 30 minutes postdose in children with attention-deficit/hyperactivity disorder (ADHD).

***Methods:*** This randomized, double-blind, two-treatment, two-sequence, placebo-controlled crossover pilot study enrolled subjects aged 6–12 years with ADHD and ADHD-Rating Scale-5 scores of ≥90th percentile for sex and age. An optimized dose of 5–20 mg/day of AMPH EROS was determined during a 1-week open-label dose optimization phase based on medication history, symptom control, and tolerability. Subjects completed a practice laboratory classroom then received 1 day of double-blind active drug or placebo each in random sequence during two double-blind laboratory classroom days. Subjects completed the first double-blind laboratory classroom, returned to open-label drug for 5 days, and then crossed over on day 6 during a second double-blind laboratory classroom. Double-blind dose was fixed at AMPH EROS 15, 17.5, or 20 mg. The primary end point was change from predose in the Swanson, Kotkin, Agler, M-Flynn, Pelham-Combined (SKAMP-C) Rating Scale score at 30 minutes postdose on two double-blind days. The key secondary end points were change from predose in the SKAMP-C score at 3 hours postdose for AMPH EROS compared with placebo and change from baseline Permanent Product Measure of Performance (PERMP) scores at 30 minutes and 3 hours postdose compared with placebo. Safety assessments included vital signs and adverse events (AEs).

***Results:*** Eighteen subjects were enrolled in the study (14 males and 4 females) with a mean age of 9 years. At both 30 minutes and 3 hours postdose, changes from baseline in SKAMP-C for AMPH EROS versus placebo were statistically significant (*p* < 0.01 and *p* = 0.0002, respectively). PERMP scores were not statistically significantly improved at 30 minutes postdose for AMPH EROS relative to the placebo group. PERMP scores were statistically significantly improved at 3 hours postdose for AMPH EROS relative to the placebo group (PERMP problems attempted treatment difference least-squares [LS] mean [SE] = 60.3 [12.93], *p* = 0.0003; PERMP problems correct treatment difference LS mean [SE] = 61.6 [13.16], *p* = 0.0003). AEs (>10%) during the open-label phase included upper respiratory tract infection, fatigue, upper abdominal pain, headache, decreased appetite, and affect lability.

***Conclusions:*** AMPH EROS was effective in reducing ADHD symptoms at 30 minutes postdose as indicated by SKAMP-C score improvement, although improvements in PERMP scores at 30 minutes were not statistically significant. AEs were mild or moderate and consistent with those of other extended-release amphetamines.

## Introduction

Amphetamine extended-release oral suspension (AMPH EROS; Dyanavel^®^ XR, Tris Pharma, Inc., Monmouth Junction, NJ) was approved by the U.S. Food and Drug Administration in 2015 for the treatment of attention-deficit/hyperactivity disorder (ADHD) in children aged 6–17 years (Tris Pharma, Inc. 2017).

The ion exchange Liqui*XR*™ technology utilized in AMPH EROS includes uncoated amphetamine, bound amphetamine, and coated bound amphetamine in a novel formulation designed to provide rapid release of active drug followed by a sustained extended release. The release characteristics of Liqui*XR* are programmable and allow for a customized sustained release of active drug product for up to 24 hours postdose. Mechanistically, drug particles enter the gastrointestinal (GI) tract. As positively-charged ions from GI fluids diffuse across the coating, ionically-charged drug product diffuses through the coating and into the GI fluids for absorption. As the coating is of variable thickness, some drug product takes longer to diffuse and absorb, providing for the programmable delayed drug release characteristic.

The efficacy and safety of AMPH EROS as a treatment for ADHD were established in a 2014 laboratory classroom study by Childress et al. (2018). In that study, 108 boys and girls aged 6–12 years diagnosed with ADHD were enrolled in a 5-week, open-label dose optimization phase and titrated to optimal dose (or maximum dose of 20 mg/day) of AMPH EROS. During the subsequent double-blind phase, subjects were randomized to receive either their optimal dose (10–20 mg/day) of AMPH EROS or placebo for 1 week.

Efficacy was assessed in a laboratory classroom setting on the final day of double-blind treatment using the Swanson, Kotkin, Agler, M-Flynn, and Pelham-Combined (SKAMP-C) Rating Scale and Permanent Product Measure of Performance (PERMP) math test. Safety was assessed using adverse events (AEs) and vital signs. The primary efficacy end point was change from predose SKAMP-C score at 4 hours postdose, and the secondary end points were change from predose SKAMP-C scores at intervals from 1 to 13 hours postdose. The study was completed by 99 subjects. The primary efficacy end point was met (least-squares [LS] mean treatment difference [95% CI] of −14.8 [−17.9 to −11.6], *p* < 0.0001). For key secondary efficacy end points, the onset of treatment effect occurred at the earliest time point assessed, 1 hour postdose (treatment difference LS mean [SE], −10.2 [1.61], *p* < 0.0001). The duration of efficacy persisted until the final time point at 13 hours postdose (treatment difference LS mean [SE], −9.2 [1.61], *p* < 0.0001).

At each postdose time point measured throughout the laboratory classroom day, the change from postdose SKAMP-C score was statistically significantly improved following treatment with AMPH EROS versus placebo. PERMP change scores from predose were also statistically significantly improved (*p* < 0.0001) at each time point from 1 to 13 hours postdose. This study demonstrated an onset of effect at 1 hour postdose and an extended duration of effect (up to 13 hours postdose) (Childress et al. 2018).

Behaviors that occur when there is inadequate control of early morning ADHD symptoms before the beginning of the school day can be especially taxing on the family and caregivers (Whalen et al. 2006). ADHD behavioral symptoms fluctuate throughout the day (Antrop et al. 2005) and can persist through midday and into the early evening hours. Survey data from caregivers of children with ADHD indicate that the early morning period before school is particularly vexing, with 76% of parents reporting functional impairments in the early morning associated with ADHD symptoms as “moderate” or “severe,” with a concordant 59.7% of caregivers indicating overall ADHD symptoms as “moderate” to “severe” throughout the entire day (Sallee 2015). A separate survey showed that early morning functional impairments associated with ADHD symptoms persist despite stimulant treatment (Faraone et al. 2017). Evidence collected to date indicates that oral extended-release amphetamines generally do not begin to control ADHD symptoms until at least 1 hour postdose, therefore, a therapeutic gap exists for the early morning period.

This exploratory study was designed and conducted to assess the early onset of effect of AMPH EROS.

## Methods

### Study design

**FIG. 1.**
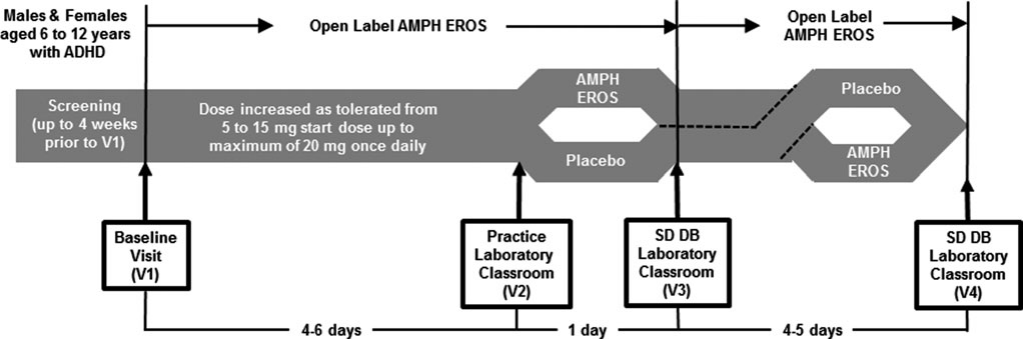
Study design. AMPH EROS, amphetamine extended-release oral suspension; SD, study drug; DB, double-blind.

This double-blind, randomized, two-period, two-treatment, placebo-controlled crossover pilot study was designed to assess the early onset (within 30 minutes postdose) efficacy and safety of AMPH EROS in reducing ADHD symptoms compared with placebo in children with ADHD aged 6–12 years. A protocol schematic is provided in Figure 1. The study consisted of five visits: screening, baseline (Visit 1), a practice classroom session (Visit 2), and double-blind study classrooms (Visits 3 and 4). Previous stimulant medication use was discontinued after Visit 1 and before initiation of study medication.

In the open-label phase, AMPH EROS was initiated once daily in the morning. Subjects who were stimulant-naive took an initial 2 mL (5 mg) per day dose of AMPH EROS. Subjects with a history of stimulant treatment started at daily doses between 2 and 6 mL (5 and 15 mg) per day based on required doses for adequate symptom control with previous stimulant medication treatment. The dose was increased as tolerated as often as daily up to a maximum dose of 8 mL (20 mg) per day or until the optimal dose was achieved as determined by the investigator. To address tolerability, doses could be decreased at the investigator's discretion. Subjects for whom the maximum study dose of 8 mL (20 mg) per day was found to be insufficient to treat their symptoms of ADHD and thus were not adequately controlled were discontinued from the study.

After Visit 2, subjects were randomized to either AMPH EROS (at optimized doses of 6, 7, or 8 mL—equivalent to 15, 17.5, or 20 mg) or matching placebo (6, 7, or 8 mL) at Visits 3 and 4. Subjects with an optimized dose of 10 mg received AMPH EROS 6 mL (15 mg) or the corresponding matching dose of placebo (6 mL). During Visits 3 and 4, subjects' attention and behaviors were rated using the SKAMP-C in the laboratory classroom and the PERMP assessment at predose, 30 minutes postdose, and 3 hours postdose. The onset of action of AMPH EROS was assessed by the change from predose in model-adjusted SKAMP-C scores at 30 minutes postdose at Visits 3 and 4, relative to placebo (primary end point). Key secondary end points included the change from predose SKAMP-C scores at 3 hours postdose and the change from predose PERMP scores at 30 minutes and 3 hours postdose, at Visits 3 and 4, as well as assessing the safety and tolerability of AMPH EROS throughout the study.

### Ethics

This study was performed in compliance with Good Clinical Practice and all applicable regulatory requirements. The study protocol was approved by an Institutional Review Board and is registered on clinicaltrials.gov under NCT03088267.

### Subjects

The study included children aged 6–12 years, who were diagnosed with ADHD through clinical assessment by a psychiatrist, psychologist, developmental pediatrician, or an experienced licensed allied health professional according to the *Diagnostic and Statistical Manual of Mental Disorders, Fifth Edition* (DSM-5) criteria (APA 2013). In addition, the subject must have scored ≥90th percentile for sex and age on the *Attention-Deficit/Hyperactivity Disorder Rating Scale, Fifth Edition* (ADHD-RS-5) (DuPaul et al. 2016) in at least one of the following categories: hyperactive-impulsive subscale, inattentive subscale, or total score before starting study drug. The ADHD diagnosis was confirmed at screening using the ADHD-RS-5. Finally, in the clinical judgment of the investigator, the subject was required to have a need for pharmacologic treatment of ADHD.

Exclusion criteria included a diagnosis of any DSM-5 active disorder (other than ADHD), with the exception of specific phobias, learning disorders, motor skill disorders, communication disorders, oppositional defiant disorder, elimination disorders, and sleep disorders. Subjects with a known history of chronic medical illnesses such as severe hypertension, untreated thyroid disease, peripheral vasculopathy, known structural cardiac conditions, serious arrhythmias, or family history of sudden death were excluded. A known history of lack of response to amphetamine was exclusionary.

### Study sites

This study was performed at a single United States based clinical site.

### Study assessments

Efficacy was assessed by trained raters using the SKAMP-C (Wigal et al. 1998; Wigal and Wigal 2006) and the PERMP (Wigal and Wigal 2006). The SKAMP-C is a 13-item, 7-point impairment scale that evaluates manifestations of ADHD in a classroom setting and includes two derivative subscales, Attention and Deportment (Wigal et al. 1998; Wigal and Wigal 2006). The PERMP is a timed written test that measures the number of math problems attempted and solved correctly in 10 minutes (Wigal and Wigal 2006). The SKAMP-C is utilized in laboratory classroom settings because it is a direct observation scale, as opposed to the Swanson, Nolan, and Pelham (Swanson 1983) or the ADHD-RS, which are rated by parents and caregivers, or by investigators based on an interview with adult caregivers. The SKAMP-C includes symptoms and behaviors that are characteristic of both ADHD and also disruptive behavior disorders more broadly. Different versions of the PERMP (with differing degrees of difficulty) were administered to subjects based on individual ability as assessed by a math pretest completed by each subject at the Baseline Visit (Visit 1). Both the SKAMP and PERMP were administered at predose and at two intervals postdose (30 minutes and 3 hours) during each laboratory classroom day (Visits 2, 3, and 4).

Safety was assessed by the incidence and severity of treatment-emergent AEs (TEAEs), which were monitored and reported throughout the study. The Columbia Suicide Severity Rating Scale (C-SSRS; The Columbia Lighthouse Project) was administered at each study visit (The Columbia Lighthouse Project 2016). Blood pressure and pulse were measured at each visit, and weight was assessed at Visit 4. Potentially clinically significant vital sign values were defined as follows: for mean systolic and diastolic blood pressure, any postbaseline value >95th percentile or any increase from baseline of ≥20 mmHg; for mean pulse, any postbaseline value >110 bpm or any increase from baseline ≥20 bpm.

### Statistical analysis

All efficacy analyses were based on the intent-to-treat population, defined as randomized subjects who received at least one dose of double-blind study drug and who had at least one postdose assessment of the primary efficacy variable at both Visits 3 and 4. All safety analyses were performed based on the safety population, defined as all subjects who received at least one dose of open-label study treatment.

The primary efficacy end point was change from predose in the model-adjusted average of SKAMP-C score at 30 minutes postdose. The treatment difference between AMPH EROS and placebo was estimated using LS means from a mixed-effects repeated-measures model with sequence, period, and treatment as fixed effects and subject within sequence as a repeated effect. Key secondary end points were change from predose SKAMP-C score at 3 hours postdose for AMPH EROS compared with placebo and change from predose PERMP score for AMPH EROS compared with placebo at 30 minutes and 3 hours postdose during the double-blind laboratory school day (Visits 3 and 4). Hypothesis testing was two sided and performed at the 5% significance level. Wherever applicable, two-sided confidence intervals (CIs) with a confidence coefficient of 95% were presented. All *p*-values ≤0.05 were considered as statistically significant.

All efficacy comparisons from the mixed-effects repeated-measures model were based on Type III tests. In the case of substantial non-normality, the normality-based analyses were carried out on the ranked scores instead of the actual values. The normality assumption was assessed for the primary efficacy variable using residual plot. When CIs were presented, they were two sided with a confidence coefficient of 95%. The effect size used in the efficacy analyses was calculated as the LS mean difference divided by the square-root of the mean-squared error.

All safety data were analyzed descriptively by treatment group. Assuming an effect size of 1.00 between AMPH EROS and placebo and ∼15 subjects completing double-blind treatment, this study had greater than 90% power at an α = 0.05 (two sided) to detect a treatment effect. To allow for an estimated 15% potential dropout rate, the targeted study enrollment was 18 subjects.

## Results

### Disposition and baseline characteristics

Study enrollment included 18 subjects, all of whom were enrolled in the safety population. All 18 subjects received double-blind AMPH EROS and placebo in the randomized crossover at Visits 3 and 4. Overall subject characteristics are shown in Table 1. More boys (77.8%) than girls (22.2%) participated in the study. The mean age of the enrolled subjects was 9.0 years. The study population was predominantly White (88.9%) and had a Combined-ADHD presentation (83.3%). The study yielded nine clinically evaluable subjects in each treatment sequence. One subject in the study population was naive to ADHD treatment with stimulant medication.

**Table 1.** Patient Characteristics

*Characteristic*	*Sequence AMPH EROS/Placebo (*n* = 9)*	*Sequence Placebo/AMPH EROS (*n* = 9)*	*Total (*n* = 18)*
Sex, *n* (%)			
Male	7 (77.8)	7 (77.8)	14 (77.8)
Female	2 (22.2)	2 (22.2)	4 (22.2)
Age, years			
Mean (SD)	8.8 (2.05)	9.2 (1.39)	9.0 (1.71)
Median	10.0	10.0	10.0
Range (min, max)	6, 11	6, 10	6, 11
Race, *n* (%)			
White	8 (88.9)	8 (88.9)	16 (88.9)
Black	1 (11.1)	1 (11.1)	2 (11.1)
Other^a^	0	0	0
Ethnicity, *n* (%)			
Hispanic/Latino	1 (11.1)	4 (44.4)	5 (27.8)
Non-Hispanic/Latino	8 (88.9)	5 (55.6)	13 (72.2)
ADHD type, *n* (%)			
Predominantly inattentive	2 (22.2)	1 (11.1)	3 (16.7)
Predominantly hyperactive/impulsive	0	0	0
Combined	7 (77.8)	8 (88.9)	15 (83.3)

Intent-to-treat population.

^a^ Race designation of “other” includes Asian, Native Hawaiian, and biracial.

ADHD, attention-deficit/hyperactivity disorder; AMPH EROS, amphetamine extended-release oral suspension; SD, standard deviation; SE, standard error.

### Efficacy assessments

**FIG. 2.**
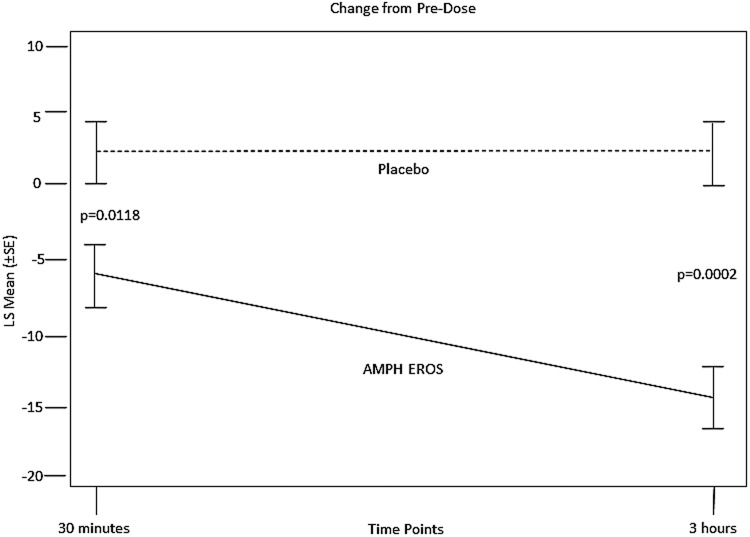
SKAMP-C scale score change from predose: double-blind treatment period (ITT population). The curves are compared using the *p*-value of the treatment effect. Treatment comparison was assessed using a linear model with sequence (Placebo/AMPH EROS, AMPH EROS/Placebo), period (Visit 3, Visit 4), treatment (AMPH EROS, Placebo), time point (30 minutes, 3 hours), the interaction term treatment × time point as fixed effects, and subject within sequence as a repeated effect with a compound symmetry correlation structure. AMPH EROS, amphetamine extended-release oral suspension; ITT, intent-to-treat; LS mean, least-squares mean; SE, standard error; SKAMP-C, Swanson, Kotkin, Agler, M-Flynn, Pehlan-Combined rating scale.

The primary efficacy end point (change from predose in the model-adjusted SKAMP-C score observed at 30 minutes postdose relative to the placebo group) was met (LS mean treatment difference [SE], −8.6 [3.01], *p* < 0.0118) (Table 2). At 3 hours, the change in SKAMP-C scores from predose was reduced by 14.3 points for subjects with AMPH EROS and increased by 2.9 points following placebo administration, a statistically significant difference (LS mean treatment difference [SE], −17.2 [3.65], *p* = 0.0002, with an effect size of −1.5743) (Fig. 2). PERMP scores (problems attempted and problems correct) were not statistically significantly improved at 30 minutes postdose for AMPH EROS relative to the placebo group (PERMP problems attempted treatment difference LS mean [SE] = 17.3 [11.01], *p* = 0.1361; PERMP problems correct treatment difference LS mean [SE] = 18.8 [11.19], *p* = 0.1128). PERMP scores (problems attempted and problems correct) were statistically significantly improved at 3 hours postdose for AMPH EROS relative to the placebo group (PERMP problems attempted treatment difference LS mean [SE] = 60.3 [12.93], *p* = 0.0003; PERMP problems correct treatment difference LS mean [SE] = 61.6 [13.16], *p* = 0.0003).

**Table 2.** Summary and Analysis of Swanson, Kotkin, Agler, M-Flynn, Pelham-Combined Scale at 30 Minutes Postdose

	*Treatment*		
*Time point statistic*	*AMPH EROS (*n* = 18)*	*Placebo (*n* = 18)*	*Treatment difference AMPH EROS-Placebo (*n* = 18)*
Predose			
Mean (SD)	25.4 (10.06)	23.8 (11.59)	
Median	25.0	23.0	
Range (min, max)	13, 51	3, 52	
30 Minutes postdose			
Mean (SD)	19.4 (12.37)	26.3 (10.00)	−6.9 (11.95)
Median	14.0	27.5	−5.0
Range (min, max)	5, 51	8, 43	−31, 17
LS, mean (SE)	19.4 (2.67)	26.3 (2.67)	−6.9 (2.68)
95% CI	13.72 to 25.05	20.67 to 32.00	−12.62 to −1.27
Effect size			−0.8650
*p*			0.0195
Change at 30 minutes postdose			
Mean (SD)	−6.1 (9.01)	2.5 (10.55)	−8.6 (12.47)
Median	−8.0	0.5	−8.5
Range (min, max)	−20, 14	−24, 18	−33, 17
LS, mean (SE)	−6.1 (2.29)	2.5 (2.29)	−8.6 (3.01)
95% CI	−10.91 to −1.20	−2.36 to 7.36	−14.91 to −2.17
Effect size			−0.9463
*p*			0.0118

ADHD, attention-deficit/hyperactivity disorder; AMPH EROS, amphetamine extended-release oral suspension; CI, confidence interval; LS, least-squares; SD, standard deviation; SE, standard error.

### Safety assessments

The mean length of exposure to study medication was 12.2 days and ranged from 11 to 13 days. The mean dose during the double-blinded classroom activity portion of the study was 15.8 mg/day, and the mean dose during the entire study was 13.3 mg/day.

During the open-label phase, 13 (72.2%) of subjects reported ≥1 TEAE. All TEAEs were considered mild (six subjects; 33.3%) or moderate (seven subjects; 38.9%) in severity. The most common TEAEs were upper respiratory tract infection (four subjects; 22.2%), and fatigue (three subjects; 16.7%). There were two subjects (11.1%) who reported decreased appetite and no reports of insomnia. No serious AEs or AEs leading to premature withdrawal were reported, and none of the subjects reported any occurrence of suicidal ideation or behavior on the C-SSRS during the study. Potentially clinically significant vital sign values during the double-blind portion of the study are summarized in Table 3. There were no clinically meaningful trends in safety reported in the study.

**Table 3.** Potentially Clinically Significant Vital Sign Values: Double-Blind Phase

		*Treatment*		
*Parameter*	*Criteria*	*AMPH EROS/Placebo (*n = *9)*	*Placebo/AMPH EROS (*n = *9)*	*Total (*N = *18)*
Systolic blood pressure	Postbaseline value >95th percentile	0	0	0
	Increase from baseline ≥20 mmHg	0	0	0
Diastolic blood pressure	Postbaseline value >95th percentile	2 (22.2)	0	2 (11.1)
	Increase from baseline ≥20 mmHg	1 (11.1)	0	1 (5.6)
Pulse	Postbaseline value >110 bpm	4 (44.4)	1 (11.1)	5 (27.8)
	Increase from baseline ≥25 bpm	3 (33.3)	2 (22.2)	5 (27.8)

AMPH EROS, amphetamine extended-release oral suspension; bpm, beats per minute.

## Discussion

AMPH EROS provided treatment efficacy in children with ADHD as early as 30 minutes postdose. Coupled with the results of the pivotal laboratory classroom study (Childress et al. 2018), AMPH EROS has shown an efficacy profile consistent with several other extended-release stimulants using a variety of formulations, with efficacy demonstrated through 13 hours postdose. Children and adolescents with ADHD often experience symptoms that interfere with functioning from the time of awakening in the before-school and early morning period, through midday, and into the early evening hours. For most children, stimulant medications are highly effective in reducing the core symptoms of ADHD as part of a comprehensive treatment plan (Pliszka et al. 2007). However, even with currently available stimulants, a significant proportion of children and adolescents remain inadequately treated. Caregivers and family members report significant emotional and functional impacts, specifically in the early morning hours, despite therapy with stimulant medication (Sallee 2015; Faraone et al. 2017). In addition, most children and adolescents require multiple doses or combinations of immediate-release and extended-release formulations (Swanson et al. 2003; Ahmed and Aslani 2013; Gajria et al. 2014) to obtain an effective treatment response. The ideal product profile for psychopharmacologic treatment of ADHD would be a single dose agent that has a rapid onset of therapeutic effect with an extended duration of effect that is sustained into the early evening (Pliszka et al. 2007). Although duration of effect (13 hours) of AMPH EROS was demonstrated in the Childress et al. (2018) study, this study was only designed to test onset of effect. This study did demonstrate an onset of effect from AMPH EROS at 30 minutes using the SKAMP-C as the primary end point; however, the full effect was not seen until later, as evidenced by the smaller effect size at the 30-minute interval compared with the larger effect size noted at 3 hours postdose. In addition, PERMP scores showed statistical separation at 3 hours but not at 30 minutes.

Taste and texture of crushed medications can influence a child's willingness to swallow the medication (Beck et al. 2005; van Riet-Nales et al. 2016). Oral liquid formulations may offer a therapeutic advantage over tablets and capsules in terms of low dosing volume, flexibility in dosing, and ease of swallowing, which are of particular importance in pediatric patients in general and pediatric patients with ADHD in particular (Beck et al. 2005; Meltzer et al. 2006; van Riet-Nales et al. 2016). AMPH EROS, when titrated to optimal doses that control symptoms, may provide an effective treatment option in this population.

This study has several limitations that impact the overall generalizability of the evidence presented. For example, the efficacy result profile was mixed. Although the SKAMP-C scores were statistically significantly improved at 30 minutes postdose compared with placebo, the PERMP scores were not statistically significantly improved at the 30-minute postdose time point. The lack of statistical significance in the PERMP measurement at 30 minutes postdose may be reflective of the overall PK profile of AMPH EROS or it may show an operational limitation of the study as the PERMP was administered to young children very early in the morning. The small sample size may have resulted in underpowering this part of the analysis. In addition, as this was a pilot study with a small sample size, the application of results across a larger and more heterogeneous patient group is limited. Furthermore, the design was enriched as subjects with ADHD who had a known lack of response to amphetamine medication were excluded. Collectively, these limitations have a direct impact on interpretation of treatment effect sizes (may appear higher) and general tolerability (AEs may appear lower).

## Conclusions

In this study, the primary efficacy end point, change from predose in the model-adjusted SKAMP-C score at 30 minutes postdose, showed a statistically significant difference in favor of AMPH EROS compared to placebo. The data presented in this study support the suggestion of the early onset efficacy of AMPH EROS as a treatment option for children with ADHD.

## Clinical Significance

The early morning is a particularly busy and often challenging time of day for children and adolescents with ADHD, as well as for their parents, families, and caregivers. Despite the numerous psychopharmacologic treatment options available to clinicians, optimized treatment of symptoms of ADHD in children ages 6–17 years remains highly individualized and often difficult to attain. An ideal profile for a psychopharmacologic treatment of ADHD symptoms in this patient group includes a relatively rapid onset of effect postdose, with a duration of effect that provides efficacy through the morning and afternoon and into the early evening hours. The data provided in this article, combined with earlier efficacy and safety data, suggest that AMPH EROS may provide a treatment effect as early as 30 minutes after administration.
